# Quantifying the Collision Dose in Rugby League: A Systematic Review, Meta-analysis, and Critical Analysis

**DOI:** 10.1186/s40798-019-0233-9

**Published:** 2020-01-22

**Authors:** Mitchell Naughton, Ben Jones, Sharief Hendricks, Doug King, Aron Murphy, Cloe Cummins

**Affiliations:** 1 School of Science and Technology, University of New England, Armidale, NSW Australia; 2 Carnegie Applied Rugby Research (CARR) centre, Institute for Sport Physical Activity and Leisure, Leeds Beckett University, Leeds, UK; 3 Leeds Rhinos Rugby League club, Leeds, UK; 4 England Performance Unit, The Rugby Football League, Leeds, UK; 5 Division of Exercise Science and Sports Medicine, Department of Human Biology, Faculty of Health Sciences, University of Cape Town, Cape Town, South Africa; 6 Health through Physical Activity, Lifestyle and Sport Research Centre (HPALS), Faculty of Health Sciences, The University of Cape Town, Cape Town, South Africa; 7 Sports Performance Institute New Zealand (SPRINZ), Faculty of Health and Environmental Science, Auckland University of Technology, Auckland, New Zealand; 8 School of Sport, Exercise and Nutrition, Massey University, Palmerston North, New Zealand; 9 National Rugby League, Sydney, Australia

**Keywords:** Global Positioning system, Microtechnology, Rugby, Tackle

## Abstract

**Background:**

Collisions (i.e. tackles, ball carries, and collisions) in the rugby league have the potential to increase injury risk, delay recovery, and influence individual and team performance. Understanding the collision demands of the rugby league may enable practitioners to optimise player health, recovery, and performance.

**Objective:**

The aim of this review was to (1) characterise the dose of collisions experienced within senior male rugby league match-play and training, (2) systematically and critically evaluate the methods used to describe the relative and absolute frequency and intensity of collisions, and (3) provide recommendations on collision monitoring.

**Methods:**

A systematic search of electronic databases (PubMed, SPORTDiscus, Scopus, and Web of Science) using keywords was undertaken. A meta-analysis provided a pooled mean of collision frequency or intensity metrics on comparable data sets from at least two studies.

**Results:**

Forty-three articles addressing the absolute (*n*) or relative collision frequency (*n* min^−1^) or intensity of senior male rugby league collisions were included. Meta-analysis of video-based studies identified that forwards completed approximately twice the number of tackles per game than backs (*n* = 24.6 vs 12.8), whilst ball carry frequency remained similar between backs and forwards (*n* = 11.4 vs 11.2). Variable findings were observed at the subgroup level with a limited number of studies suggesting wide-running forwards, outside backs, and hit-up forwards complete similar ball carries whilst tackling frequency differed. For microtechnology, at the team level, players complete an average of 32.7 collisions per match. Limited data suggested hit-up and wide-running forwards complete the most collisions per match, when compared to adjustables and outside backs. Relative to playing time, forwards (*n* min^−1^ = 0.44) complete a far greater frequency of collision than backs (*n* min^−1^ = 0.16), with data suggesting hit-up forwards undertake more than adjustables, and outside backs. Studies investigating *g* force intensity zones utilised five unique intensity schemes with zones ranging from 2–3 *g* to 13–16 *g*. Given the disparity between device setups and zone classification systems between studies, further analyses were inappropriate. It is recommended that practitioners independently validate microtechnology against video to establish criterion validity.

**Conclusions:**

Video- and microtechnology-based methods have been utilised to quantify collisions in the rugby league with differential collision profiles observed between forward and back positional groups, and their distinct subgroups. The ball carry demands of forwards and backs were similar, whilst tackle demands were greater for forwards than backs. Microtechnology has been used inconsistently to quantify collision frequency and intensity. Despite widespread popularity, a number of the microtechnology devices have yet to be appropriately validated. Limitations exist in using microtechnology to quantify collision intensity, including the lack of consistency and limited validation. Future directions include application of machine learning approaches to differentiate types of collisions in microtechnology datasets.

## Key Points

- Video- and microtechnology-based methods have been employed to quantify collision (including tackle and ball carry) frequency and intensity with position-specific differences observed.
- A number of microtechnology devices that purport collision detection capacity have yet to be appropriately validated, as such practitioners need to be aware of these limitations when choosing and utilising such devices.
- There are considerable gaps in the understanding of effectively quantifying collisions in the rugby league, which may be explored by applying machine learning methods to microtechnology datasets.

## Introduction

Rugby league is an invasion contact sport played in over 14 countries, in which senior male rugby league consists of two 40-min halves [1]. A match is contested by 13 players on two opposing teams. The fundamental goal of rugby league is to score more points than the opposition team, and this can be achieved by scoring a try (i.e. grounding the ball beyond the oppositions try line) or kicking a goal (i.e. a drop goal, penalty kick, or try conversion) [2]. Whilst the demands of the rugby league are specific to the respective competition [3], playing level [4], and positional group [5], the game typically involves intermittent periods of low-intensity exercise (such as walking or jogging), interspersed with periods of high-intensity efforts (such as accelerations, decelerations, running, and sprinting) [6–8].

The Global Positioning System (GPS) is an accurate satellite-based navigational technology that was first launched in 1978 [9–11]. Commercial GPS devices were first utilised within sporting contexts in 1997 [12] . Upon their introduction, these devices sampled at 1 Hertz (Hz) with limited accompanying software [12]. Such devices have, however, evolved over time to include higher sampling rates (e.g. 5 or 10 Hz) and custom proprietary local software and cloud-based computing [13]. Alongside this evolution, additional inertial sensors such as accelerometers, gyroscopes, and magnetometers have been incorporated into these devices [14]. These sensors provide information on the instantaneous rate of accelerations in the *x*-, *y-,* and *z*-axis (anteroposterior, mediolateral, and vertical), as well as yaw, pitch, and roll, and unit orientation in relation to the earth’s magnetic poles [9]. The combination of GPS and imbedded inertial sensors is referred to as a microtechnology device. Utilising microtechnology in sporting contexts, research has examined a variety of variables including work rate patterns, movement profiles, and the peak locomotor demands of training and competition in sports such as soccer [15–18], Australian rules [16, 19, 20], rugby union [21, 22], and rugby league [4, 14, 23, 24].

Microtechnology devices were first introduced into the professional rugby league via the National Rugby League (NRL) and European Super League (SL) in 2009 and 2010, respectively. Since their introduction, there has been an increase in the research utilising these devices to monitor match-play and training demands. A PubMed search of all studies published between 2009 and 2019 using the terms ‘Rugby League AND GPS’ identified an increase from one article in 2010, to 19 published in 2018 and 2019. The locomotor demands of the rugby league have been previously described in detail [4, 25]. Additionally, the rugby league is characterised by collisions between teammates, opponents, and the playing surface [26]. These collisions typically occur between the tackler(s) and the ball carrier during the tackle event and have been reported to lead to soreness and muscle damage which compromises muscle integrity, attenuates force generation capacity, and has the potential to delay athletic recovery [27]. Additionally, the vast majority (~ 94%) of match-related injuries in the professional rugby league are tackle related [28]. Furthermore, dominance in collision events has been shown to relate to match performance (i.e. match outcome) from both attacking and defending perspectives [29–31]. Given the apparent importance of offensive (e.g. ball carries) and defensive collisions (e.g. tackles) to match outcome and player health and wellbeing, it is imperative for coaches and practitioners to specifically monitor the collision demands of both training and competition activities.

Historically, quantification of the volume and intensity of collisions experienced (i.e. ‘dose*’*) by rugby league athletes have occurred via tallies of tackles and ball carries, and through qualitative examination of the perceived dominance in collisions from analysis of video footage [32, 33]. While this process can provide a rich source of contextual data, it is often labour and resource intensive and may be prone to the subjective biases of the video analyst [31]. Furthermore, the time taken to analyse these activities can be problematic due to the limited turn-around between matches and training sessions. To address this, microtechnology has been utilised to automate the assessment of impacts and collisions based on changes in unit orientation through proprietary algorithms [34, 35]. Indeed, a number of microtechnology devices now have automated impact and collision detection capacity from companies including Catapult Sports (Catapult Sports, Melbourne, Victoria, Australia), STATSports (STATSports, Newry, Northern Ireland) and GPSports (GPSports, Canberra, Australian Capital Territory, Australia). Similarly, microtechnology has been utilised to quantify collision intensity through summating the forces acting upon the accelerometer into *g* force intensity zones. However, the validity of these approaches in quantifying both collision frequency and intensity through microtechnology is unclear. Furthermore, an understanding of the collision dose experienced in the male senior rugby league has yet to be fully elucidated. Given the rapid commercial development in this area, and the importance of quantifying collisions, this systematic review characterises the dose of collisions experienced within senior rugby league training and match activities and examines the utility of microtechnology devices in quantifying collisions. Therefore, in relation to the male senior rugby league, the specific aims of this review were to (1) evaluate the methods used to describe the relative and absolute frequency of collisions, (2) evaluate the methods used to describe the relative and absolute intensity of collisions, (3) collate the collision demands of match-play and training, and (4) critically examine the literature and provide recommendations on the monitoring of collision loads in the rugby league.

## Methods

### Design

Studies investigating the collision dose experienced by male senior rugby league athletes (i.e. athletes over 18 years of age) in training and match activities or game simulation were eligible for inclusion. A systematic search of electronic databases (PubMed, SPORTDiscus, Scopus, and Web of Science) was conducted from January 1990 to March 2019. The search strategy combined terms for collisions (‘tackl*’, OR ‘collision’, OR ‘impact*’), AND dose (‘frequency’, OR ‘intensity’, OR ‘demands’), AND rugby league (rugby*, OR ‘rugby league). Any study that examined the frequency, intensity or the type of collision (such as impacts, collisions, tackles, ball carries) in a quantitative manner was included.

### Selection of Studies

**Fig. 1:**
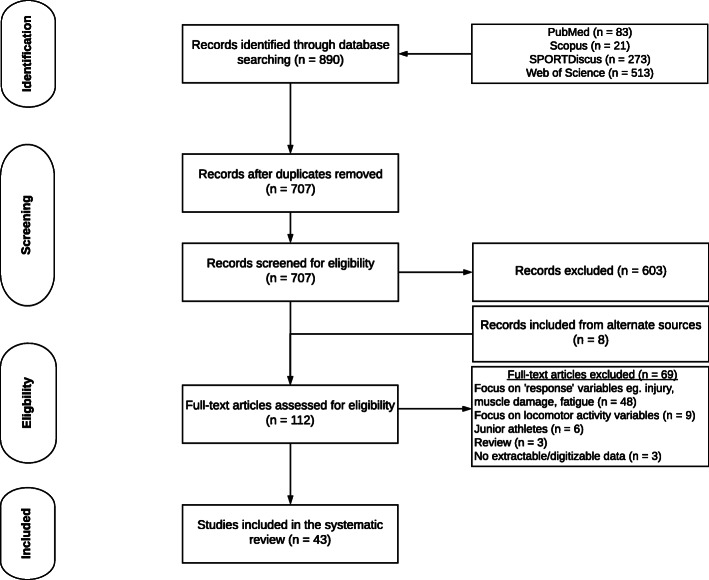
Selection process of eligible studies in the systematic review

Following the elimination of duplicate manuscripts, the search results were parsed for eligibility by examination of the title and abstract by one of the researchers (MN). References that could be eliminated by title or abstract examination were removed and the remaining studies were screened by two researchers (MN, CC) against the eligibility criteria. Screening occurred via a customised spreadsheet, and there were no disagreements in the included studies between researchers. Reviewers were not masked to the names of authors or the title of publications. Abstracts and conference papers from annual meetings were not included due to not meeting the rigour of outcome measures. In instances where journal articles contained insufficient information, attempts were made to contact the authors in order to obtain further details, with one paper being excluded due to data not being made available to the authors on request [36]. Papers from all languages were included but were excluded if translation to English could not be made. Reference lists of papers included in the final analysis were screened for inclusion of other potentially eligible papers as ‘included from alternate sources’ (Fig. 1).

### Data Extraction

Data relating to the participant characteristics (i.e. age, height, body mass, and competition level), the method used to quantify collisions (i.e. video or microtechnology), microtechnology device (i.e. model, manufacturer, recording frequency, presence of inertial sensors), collision characteristics, frequency of collisions, and the intensity of collisions were extracted. Collision characteristics included what was reported with respect to impacts, collisions, or differentiated into player tackle or ball carry into contact. The frequency of collisions was extracted as the absolute number (*n*) per match/training session at either the team, season, or competition level. Furthermore, collisions relative to playing or training time (*n* min^−1^) were extracted. The intensity of collisions were extracted from studies which provided mild, moderate, or heavy descriptors based on the nature of the event [31], with these categories based on microtechnology data. The absolute and relative frequencies within these classifications were also extracted from collated data. Similarly, the relative and absolute frequency of collision forces (*g* forces) were divided into 4 to 6 different zones (zones 1–6) ranging from < 5 to > 10.1 *g* [37, 38]. Each zone was linked to a qualitative description ranging from light impacts and change of direction to severe impacts and player collisions [4]. Velocity and acceleration into contact were also extracted as a collision pseudo-intensity metric. Data which were only available in graphical form were extracted by digitising of the figures with WebPlotDigitizer [39].

### Assessment of Methodological Quality

The quality of the included studies was independently assessed using the modified assessment scale of Downs and Black [40]. Of the 27 criteria, 12 questions were examined that logically applied to the study designs utilising microtechnology or video analysis in sport. These criteria questions reflected subscales that relate to external validity (numbers 11, 12), internal validity (16, 18, 20), and reporting (1–4, 6, 7, 10). Assessment of quality was completed by two of the authors (MN, CC). No studies were eliminated, and no additional subgroup analysis was undertaken on the basis of methodological quality.

### Statistics

All data are presented as mean or mean ± standard deviation (SD) unless otherwise stated. Where possible, data that were published as mean and associated confidence intervals were transformed to SD [24, 41, 42] utilising methods outlined in the Cochrane handbook [43]. Where this was not possible due to insufficient information (*n* = 2 studies [43, 44]), data were reported as mean and relevant confidence intervals. Studies were included in the review if they reported the number of player files or the number of participants. Meta-analyses (Review Manager, Version 5.3) were conducted to provide a pooled mean with 95% confidence intervals for collision dose of the groups and subgroups for which comparable data were extracted from at least two similar studies. Meta-analysis was not undertaken on grouped or sub-grouped data when there were insufficient data to compare between studies for a given group or subgroup comparison. For consistency, studies were entered into the meta-analysis if they reported the number of players as the sample size, and SD or SE was reported. When studies reported data from different cohorts within the same study, these were treated as data from separate studies [45]. Heterogeneity of studies within- and between-subgroups was assessed via chi-squared (Chi^2^), tau-squared (Tau^2^), and *I*-squared (*I*^2^) statistics [43]. An *I*^2^ of 0–40%, 40–75%, and > 70% was considered low, moderate, and high heterogeneity respectively [43]. The following variables were included in the meta-analysis; positional group, absolute collision frequency (*n*), relative collision frequency (*n* min^−1^), absolute collision intensity (mild/moderate/heavy), and the type of collision reported (tackle/ball carry/collision/impact/total).

## Results

### Identification and Selection of Studies

The original search captured 890 papers (Fig. 1). After the removal of duplicates and screening, 43 studies were included in the systematic review [1, 7, 8, 16, 23, 24, 26, 28, 31–33, 35, 36, 38, 41, 42, 44, 46–71].

### Methodological Quality

There were 43 studies that met the inclusion criteria. The methodological quality of these studies was moderate to good, with scores ranging from 6 to 11 across the 11 items that were assessed (see Table 1).

**Table 1:** Methodological quality assessment of the included studies (Downs and Black [40])

Study	Question number											Total score
	1	2	3	6	7	10	11	12	16	18	20	
Austin et al. [46]	1	1	0	1	0	0	0*	0	1	1	1	6
Cummins and Orr [44]	1	1	0	1	0	1	1	1	1	1	1	10
Cummins and Orr [26]	1	1	1	1	1	1	0*	0	1	1	1	9
Dempsey et al. [8]	1	1	1	1	1	0	0*	1	1	1	1	9
Evans et al. [69]	1	1	1	1	1	1	0*	0	1	1	1	9
Fletcher et al. [70]	1	1	1	1	1	1	0*	0	1	1	1	9
Gabbett et al. [31]	1	1	1	1	1	0	0*	0	1	1	1	8
Gabbett and Ryan [71]	1	1	1	1	1	1	0*	0	1	1	1	9
Gabbett [33]	1	1	1	1	1	1	0*	0	1	1	1	9
Gabbett [23]	0	1	1	1	1	1	0*	0	1	1	1	8
Gabbett [47]	1	1	1	1	1	1	1	1	1	1	1	11
Gabbett [48]	1	1	1	1	1	1	1	1	1	1	1	11
Gabbett and Hulin [36]	1	1	0	1	1	1	1	1	1	1	1	10
Gabbett et al. [49]	1	1	1	1	1	0	0*	0	1	1	1	8
Gabbett et al. [50]	1	1	1	1	1	0	0*	0	1	1	1	8
Gabbett et al. [24]	1	1	1	1	1	1	0*	0	1	1	1	9
Gabbett et al. [51]	1	1	1	1	1	1	0*	0	1	1	1	9
Gabbett et al. [52]	1	1	1	1	1	1	0*	0	1	1	1	9
Gabbett et al. [53]	1	1	1	1	1	1	0*	0	0	1	1	8
Hulin and Gabbett [54]	1	1	1	1	1	1	0*	1	1	1	1	10
Hulin et al. [35]	1	1	1	1	1	1	0*	0	1	1	1	9
Hulin et al. [55]	1	1	1	1	1	1	0*	1	1	1	1	10
Johnston et al. [56]	1	1	1	1	1	1	0*	0	1	1	1	9
Kempton et al. [57]	1	1	1	1	1	1	0*	0	1	1	1	9
Kempton et al. [58]	1	1	1	1	1	1	0*	0	1	1	1	9
Kempton et al. [42]	1	1	1	1	1	1	0*	1	1	1	1	10
King et al. [41]	1	1	0	1	1	1	1	1	1	1	1	11
King et al. [28]	1	1	0	1	1	1	0*	0	1	1	1	9
Lovell et al. [59]	1	1	1	1	1	1	0*	0	1	1	1	9
McLellan and Lovell [60]	1	1	1	1	1	1	0*	0	1	1	1	9
McLellan et al. [38]	1	1	1	1	1	1	0*	0	1	1	1	9
Murray et al. [61]	1	1	1	1	1	1	0*	0	1	1	1	9
Oxendale et al. [62]	1	1	1	1	1	1	0*	0	1	1	1	9
Sirotic et al. [32]	1	1	1	1	1	1	0*	0	1	1	1	9
Sirotic et al. [63]	1	1	1	1	1	1	0*	0	1	1	1	9
Speranza et al. [64]	1	1	1	1	1	1	0*	0	1	1	1	9
Sykes et al. [65]	1	1	0	1	1	0	0*	0	1	1	1	7
Twist et al. [66]	1	1	1	1	1	1	0*	0	1	1	1	9
Varley et al. [16]	1	1	0	1	1	1	0*	0	1	1	1	9
Weaving et al. [67]	1	1	1	1	1	1	0*	0	1	1	1	9
Weaving et al. [7]	1	1	1	1	1	1	0*	0	1	1	1	9
Woods et al. [1]	1	1	0	1	1	1	1	1	1	1	1	10
Woods et al. [68]	1	1	0	1	1	1	1	1	1	1	1	10

1. Is the hypothesis/aim clearly described? 2. Are the main outcomes to be measured clearly described in the introduction/methods sections? 3. Are the characteristics of the participants included in the study clearly described? 6. Are the main findings of the study clearly described? 7. Does the study provide estimates of the variability in the data for the main outcome? 10. Have *p* values/effect sizes for the main outcome been reported? 11. Were the subjects who were asked to participate representative of the wider population of interest? 12. Were the subjects who were prepared to participate representative of the wider population of interest? 16. Were any of the results based on ‘data dredging’, was this made clear? 18. Were the statistical tests used for the main outcomes appropriate? 20. Were the main outcome measures used accurate and reliable? *Unable to determine

### Study Characteristics

Collectively, 1384 participants were examined in 39 of the 43 included studies. From these 39 studies, participants were drawn from teams in the NRL, SL, Australian State Leagues (predominantly the Queensland Cup [QCup]), International, Australian Under 20’s National Youth Competition (NYC), and amateur competitions (see Table 2). The remaining four studies reported on participants with collisions analysed via video footage at the competition level [1, 28, 41, 68].

**Table 2:** Study characteristics of the included studies

Study	Method of collision capture	Level of competition	No. of participants (*n* = )
Austin et al. [46]	Video	NRL	15
Cummins and Orr [44]	Video and microtechnology	NRL	NR (video)
			10 (microtechnology)
Cummins and Orr [26]	Video and microtechnology	NRL	26
Dempsey et al. [8]	Video	International	57
Evans et al. [69]	Microtechnology	Super League	33
Fletcher et al. [70]	Video	Super League	31
Gabbett et al. [31]	Video and microtechnology	NRL	30
Gabbett and Ryan [71]	Video	NRL	22
		QCup	17
Gabbett [33]	Video	Local	8
Gabbett [23]	Microtechnology	NRL	24
		NYC	11
Gabbett [47]	Microtechnology	QCup	182
Gabbett [48]	Microtechnology	QCup	104
Gabbett et al. [49]	Video	NRL	51
Gabbett et al. [50]	Video	NRL	58
Gabbett et al. [24]	Microtechnology	NRL	30
Gabbett et al. [51]	Microtechnology	NRL	22
Gabbett and Seibold [52]	Microtechnology	QCup	32
Gabbett et al. [53]	Microtechnology	NRL	38
Hulin and Gabbett [54]	Microtechnology	QCup	77
Hulin et al. [35]	Video and microtechnology	NRL	8
Hulin et al. [55]	Microtechnology	NRL	31
Johnston et al. [56]	Microtechnology	International Student Competition	7
Kempton et al. [57]	Microtechnology	NRL	6
Kempton et al. [58]	Microtechnology	NRL	18
Kempton et al. [42]	Microtechnology	NRL	29 (more successful)
			25 (less successful)
King et al. [41]	Video	International	NR
		NRL	NR
King et al. [28]	Video	NRL	NR
Lovell et al. [59]	Microtechnology	NRL	32
McLellan and Lovell [60]	Video and microtechnology	NRL	22
McLellan et al. [38]	Video and microtechnology	NRL	17
Murray et al. [61]	Microtechnology	NRL	43
Oxendale et al. [62]	Microtechnology	Super League	17
Sirotic et al. [32]	Video	NRL	17
		NSWCup	22
Sirotic et al. [63]	Video	NRL	17
Speranza et al. [64]	Video	QCup	16
Sykes et al. [65]	Video	NRL	26
		Super League	52
Twist et al. [66]	Video	Super League	23
Varley et al. [16]	Microtechnology	NRL	36
Weaving et al. [67]	Microtechnology	Super League	17
Weaving et al. [7]	Microtechnology	Super League	25
Woods et al. [1]	Video	NRL	NR
		Super League	NR
Woods et al. [68]	Video	NRL	NR
		NYC	NR

Data are reported as mean ± standard deviation (SD) unless otherwise stated. *Less successful* team lost more games than it won in the more successful season, *more successful* team won more games than the less successful season, *NR* not reported, *NRL* National Rugby League, *NYC* National Youth Competition

Of the studies that compared cohorts between competition standards, one study compared cohorts from the NRL to Qcup [71], one study compared NRL cohorts to NSWCup [32], two studies compared NRL to SL [1, 65], two studies compared NRL to NYC [23, 68], and one study compared international to NRL [41]. Studies typically compared the collision dose either at the overall team level or within positional groupings. Analyses undertaken within positional groups included either two (backs and forwards [8, 23, 28, 38, 41, 47, 51, 52, 60, 62, 64, 66]) or four (hit-up forwards [props], wide-running forwards [second-rowers, locks], adjustables [full-back, five-eighth, half-back, and hooker], and outside backs [wingers and centres] [24, 26, 28, 31, 41, 49, 54, 55, 65, 69, 70]) positional groups. Of the included studies, 23 studies reported collision dose features such as the absolute frequency of collisions per match (*n*), collisions relative to a player’s time on field (*n* min^−1^), or collision intensity features that were derived from analysis of video footage (see Table 3). Furthermore, 26 studies reported features derived from microtechnology alone (see Table 4) and six studies utilised both methods to analyse features of collisions (see Tables 3 and 4). Collision frequency across a multi-game period were averaged over the number of games to provide a per match frequency in two studies which were included [46, 56]. Three studies were not included in the final analysis as data was reported across selected 5- and 10-min periods and the integrity of absolute or relative frequency data over a match could not be established from the available summary statistics or from digitising of the figures [51, 54, 57].

**Table 3:** Characteristics of collisions during match-play recorded by video notational analysis

Study	Competition (season[s])	Positional group	Type of collision recorded	Frequency of collisions (*n*=) mean (±SD)	Relative frequency of collisions (*n* min^−1^=) mean (±SD)
Austin et al. [46]	NRL (2008)	Hit-up forwards	Tackles and ball carries	33.2 (NR)	NR
		Adjustables	Tackles and ball carries	17.8 (NR)	NR
		Outside backs	Tackles and ball carries	8.2 (NR)	NR
Cummins et al. [26]	NRL (NR)	Hit-up forwards	Tackles	21.5 (6.1)	0.52 (0.09)
			Ball carries	8.9 (3.7)	0.20 (0.03)
			Tackles and ball carries	30.5 (9.6)	0.78 (0.11)
		Wide-running forwards	Tackles	20.6 (5.0)	0.39 (0.10)
			Ball carries	7.9 (3.7)	0.20 (0.10)
			Tackles and ball carries	29.8 (6.2)	0.57 (0.20)
		Adjustables	Tackles	16.7 (12.8)	0.41 (0.20)
			Ball carries	4.9 (4.6)	0.10 (0.00)
			Tackles and ball carries	21.7 (12.3)	0.49 (0.20)
		Outside backs	Tackles	7.0 (6.1)	0.08 (0.07)
			Ball carries	11.2 (2.0)	0.10 (0.02)
			Tackles and ball carries	18.3 (5.4)	0.21 (0.06)
Dempsey et al. [8]	SL (2011–2012)	Backs	Tackles	13.4 (9.5)	0.16 (0.11)
			Ball carries	11.9 (5.2)	0.15 (0.08)
		Forwards	Tackles	25.5 (8.4)	0.47 (0.23)
			Ball carries	10.5 (3.6)	0.20 (0.10)
Fletcher et al. [70]	SL (2012)	Hit-up forwards	Tackles	24.0 (13.0)	NR
			Ball carries	8.5 (5.0)	NR
			Tackles and ball carries	32.0 (15.0)	0.60 (0.30)
		Adjustables	Tackles	14.0 (12.0)	NR
			Ball carries	4.0 (4.0)	NR
			Tackles and ball carries	21.0 (12.0)	0.30 (0.30)
		Outside backs	Tackles	8.0 (10.0)	NR
			Ball carries	9.0 (4.0)	NR
			Tackles and ball carries	19.0 (9.0)	0.30 (0.10)
Gabbett and Ryan [71]	NRL (2008–2009)	Team	Tackles	24.0 (NR)	NR
Gabbett et al. [49]	NRL (2008–2010)	Hit-up forwards	Tackles (total defensive)	23.0 (21.0,25.0)*	NR
			Ball carries (total attack)	13.0 (11.0,15.0) *	NR
			Tackles and ball carries	36.0 (32.0,40.0) *	NR
		Wide-running forwards	Tackles (total defensive)	30.0 (26.0,34.0) *	NR
			Ball carries (total attack)	17.0 (13.0,21.0) *	NR
			Tackles and ball carries	47.0 (42.0,52.0) *	NR
		Adjustables	Tackles (total defensive)	19.0 (15.0,23.0) *	NR
			Ball carries (total attack)	10.0 (7.0,13.0) *	NR
			Tackles and ball carries	29.0 (26.0,32.0) *	NR
		Outside backs	Tackles	11.0 (9.0,13.0) *	NR
			Ball carries	13.0 (12.0,14.0) *	NR
			Tackles and ball carries	24.0 (22.0,27.0) *	NR
Gabbett et al. [50]	NRL (2008–2011)	Team	Tackles	17.1 (9.1)	NR
			Ball carries	8.8 (2.8)	NR
King et al. [41]	International (2008)	Game	Tackles and ball carries	620.6 (NR)	NR
		Backs	Tackles	377.0 (22.9)	NR
			Ball carries	285.7 (21.6)	NR
		Forwards	Tackles	623.0 (29.4)	NR
			Ball carries	238.1 (19.6)	NR
		Hit-up forwards	Tackles	386.5 (23.3)	NR
			Ball carries	340.9 (32.4)	NR
		Adjustables	Tackles	404.8 (23.8)	NR
			Ball carries	224.3 (26.2)	NR
		Outside backs	Tackles	208.6 (17.1)	NR
			Ball carries	434.9 (36.6)	NR
	NRL (2008)	Game	Tackles and ball carries	650.8 (NR)	NR
		Backs	Tackles	343.6 (22.5)	NR
			Ball carries	257.2 (19.8)	NR
		Forwards	Tackles	656.4 (31.2)	NR
			Ball carries	229.7 (18.7)	NR
		Hit-up forwards	Tackles	378.5 (23.6)	NR
			Ball carries	366.3 (34.1)	NR
		Adjustables	Tackles	451.0 (25.7)	NR
			Ball carries	199.1 (12.9)	NR
		Outside backs	Tackles	170.5 (15.9)	NR
			Ball carries	434.6 (37.0)	NR
King et al. [28]	NRL (NR)	Game	Tackles (completed)	590.0 (50.0)	NR
		Backs	Tackles and ball carries (completed and missed)	14.6 (7.7)	NR
		Forwards	Tackles and ball carries (completed and missed)	27.1 (8.3)	NR
McLellan and Lovell [60]	NRL (NR)	Team	Tackles	19.9 (10.5)	NR
			Ball carries	12.2 (3.6)	NR
		Backs	Tackles	10.7 (8.9)	NR
			Ball carries	11.7 (4.6)	NR
		Forwards	Tackles	26.1 (15.3)	NR
			Ball carries	13.8 (5.2)	NR
McLellan et al. [38]	NRL (NR)	Team	Tackles	14.9 (10.5)	NR
			Ball carries	10.2 (3.8)	NR
		Backs	Tackles	10.7 (8.0)	NR
			Ball carries	9.7 (3.5)	NR
		Forwards	Tackles	20.1 (11.3)	NR
			Ball carries	10.9 (4.2)	NR
Sirotic et al. [32]	NRL (2004–2005)	Team	Tackles	NR	0.25 (0.16)
			Ball carries	NR	0.15 (0.08)
	NSWCup (2004–2005)	Team	Tackles	NR	0.28 (0.16)
			Ball carries	NR	0.15 (0.08)
Sirotic et al. [63]	NRL (2004–2005)	Backs	Tackles	NR	0.12 (0.09)
			Ball carries	NR	0.11 (0.04)
		Forwards	Tackles	NR	0.41 (0.07)
			Ball carries	NR	0.25 (0.09)
Sperenza et al. [64]	QCup (2014)	Team	Tackles	18.0 (NR)	NR
		Backs	Tackles	13.2 (8.5)	NR
		Forwards	Tackles	24.3 (6.5)	NR
Twist et al. [66]	SL (2010)	Backs	Tackles	13.6 (7.9)	0.2 (0.10)
			Ball carries	11.6 (3.4)	0.1 (0.04)
			Tackles and ball carries	25.2 (8.0)	0.3 (0.10)
		Forwards	Tackles	25.5 (13.7)	0.5 (0.20)
			Ball carries	12.7 (6.1)	0.3 (0.10)
			Tackles and ball carries	38.2 (18.7)	0.7 (0.30)
Woods et al. [1]	NRL (2016)	Game	Tackles	314.3 (15.9)	NR
			Ball carries	164.3 (13.5)	NR
	SL (2016)	Game	Tackles	336.1 (11.8)	NR
			Ball carries	179.0 (8.1)	NR
Woods et al. [68]	NRL (2016)	Game	Tackles	325.0 (39.7)	NR
			Ball carries	170.2 (19.8)	NR
	NYC (2016)	Game	Tackles	283.4 (35.6)	NR
			Ball carries	147.2 (17.4)	NR

Data are reported as mean ± standard deviation (SD) unless otherwise stated. *Data are reported as mean (±95% confidence intervals) as SD was not able to be determined due to insufficient information. *Game* results for both teams involved at the game level, *NR* not reported, *NRL* National Rugby League, *NYC* National Youth Competition, *NSWCup* New South Wales Cup, *QCup* Queensland Cup, *SL* Super League, *Team* results at the individual team level

**Table 4:** Characteristics of collisions during match-play recorded by detection from microtechnology units by studies reporting mild, moderate, and heavy collisions

Study	Microtechnology provider (device)	Competition (season[s])	Group (variable)	Positional group	Type of collision recorded	Frequency of collisions (*n*=) mean (±SD)				Relative frequency of collisions (*n* min^−1^=) mean (±SD)			
						Mild	Moderate	Heavy	Total	Mild	Moderate	Heavy	Total
Gabbett et al. [31]	Catapult (minimaxX)	NRL (2008–2009)		Hit-up forwards	Collisions	2.0 (NR)	20.0 (NR)	15.0 (NR)	37.0 (NR)	0.06 (NR)	0.56 (NR)	0.41 (NR)	1.02 (NR)
				Wide-running forwards	Collisions	2.0 (NR)	12.0 (NR)	14.0 (NR)	28.0 (NR)	0.03 (NR)	0.27 (NR)	0.28 (NR)	0.59 (NR)
				Adjustables	Collisions	1.0 (NR)	14.0 (NR)	15.0 (NR)	30.0 (NR)	0.02 (NR)	0.21 (NR)	0.23 (NR)	0.45 (NR)
				Outside backs	Collisions	5.0 (NR)	10.0 (NR)	2.0 (NR)	16.0 (NR)	0.03 (NR)	0.20 (NR)	0.25 (NR)	0.48 (NR)
Gabbett [23]	Catapult (minimaxX)	NRL (NR)		Forwards	Collisions	0.2 (0.50)	10.1 (4.80)	13.0 (4.3)	23.3 (7.6)	0.01 (0.01)	0.20 (0.09)	0.27 (0.08)	0.47 (0.13)
				Adjustables	Collisions	0.5 (0.8)	6.5 (3.5)	9.4 (4.5)	16.4 (6.5)	0.01 (0.01)	0.09 (0.06)	0.13 (0.06)	0.23 (0.10)
				Outside backs	Collisions	0.2 (0.6)	4.3 (2.8)	11.9 (4.3)	16.4 (6.1)	0.01 (0.01)	0.06 (0.04)	0.15 (0.05)	0.21 (0.07)
		NYC (NR)		Forwards	Collisions	0.6 (0.8)	7.4 (6.3)	10.3 (4.7)	18.3 (10.5)	0.01 (0.01)	0.13 (0.07)	0.21 (0.08)	0.35 (0.11)
				Adjustables	Collisions	0.3 (0.5)	8.3 (6.1)	10.7 (2.9)	19.3 (6.7)	0.01 (0.01)	0.13 (0.11)	0.15 (0.04)	0.29 (0.14)
				Outside backs	Collisions	NR	6.0 (3.3)	8.8 (8.2)	14.8 (9.1)	NR	0.08 (0.03)	0.11 (0.10)	0.19 (0.10)
Gabbett [47]	Catapult (minimaxX S4)	QCup (2012)		Forwards	Collisions	NR	NR	NR	44.0 (13.0)	NR	NR	NR	0.77 (0.19)
				Adjustables	Collisions	NR	NR	NR	29.0 (9.0)	NR	NR	NR	0.35 (0.10)
				Outside backs	Collisions	NR	NR	NR	22.0 (8.0)	NR	NR	NR	0.27 (0.10)
				Hookers	Collisions	NR	NR	NR	40.0 (13.0)	NR	NR	NR	0.64 (0.16)
Gabbett [48]	Catapult (minimaxX S4)	QCup (2012)	Match-play	Team	Collisions	4.6 (4.5)	21.7 (10.5)	6.5 (3.8)	32.9 (13.7)	0.06 (0.06)	0.27 (0.13)	0.08 (0.05)	0.41 (0.17)
			Ball-in-play	Team	Collisions	4.6 (4.5)	21.7 (10.5)	6.5 (3.8)	32.9 (13.7)	0.09 (0.09)	0.44 (0.21)	0.13 (0.08)	0.67 (0.28)
Gabbett et al. [24]	Catapult (minimaxX)	NRL (NR)	Match-play	Team	Collisions	3.0 (2.3)	19.0 (4.7)	15.0 (2.3)	37.0 (5.8)	0.06 (0.02 )	0.34 (0.12)	0.26 (0.06)	0.68 (0.18)
				Hit-up forwards	Collisions	4.0 (3.7)	22.0 (11.0)	16.0 (4.9)	42.0 (15.9)	0.09 (0.10)	0.58 (0.21)	0.43 (0.12)	1.09 (0.32)
				Wide-running forwards	Collisions	4.0 (4.9)	24.0 (9.8)	17.0 (6.1)	45.0 (17.1)	0.07 (0.06)	0.39 (0.15)	0.29 (0.09)	0.76 (0.18)
				Adjustables	Collisions	4.0 (4.1)	19.0 (11.0)	11.0 (6.9)	34.0 (16.5)	0.07 (0.07)	0.33 (0.22)	0.20 (0.10)	0.58 (0.36)
				Outside backs	Collisions	2.0 (2.8)	12.0 (5.5)	14.0 (5.5)	28.0 (12.4)	0.03 (0.03)	0.17 (0.08)	0.20 (0.07)	0.38 (0.15)
			Training—repeated high intensity efforts	Team	Collisions	5.0 (6.8)	20.0 (13.5)	1.0 (1.7)	26.0 (15.2)	0.13 (0.12)	0.54 (0.42)	0.02 (0.05)	0.69 (0.47)
			Training—games	Team	Collisions	4.0 (4.5)	5.0 (4.5)	2.0 (2.2)	13.0 (9.0)	0.31 (0.27)	0.38 (0.31)	0.12 (0.13)	0.81 (1.12)
			Training—skills	Team	Collisions	8.0 (5.7)	15.0 (5.7)	3.0 (2.8)	26.0 (11.3)	0.10 (0.06)	0.20 (0.06)	0.04 (0.03)	0.29 (0.06)
Gabbett and Seibold [52]	Catapult (minimaxX S4)	QCup (NR)		Backs	Collisions	NR	NR	NR	26.0 (5.0)	NR	NR	NR	0.3 (0.1)
				Forwards	Collisions	NR	NR	NR	32 (8)	NR	NR	NR	0.7 (0.2)
Gabbett et al. [53]	Catapult (minimaxX)	NRL (NR)	Higher aerobic fitness	Team	Collisions	NR	NR	NR	45.2 (13.4)	NR	NR	NR	0.92 (0.34)
			Lower aerobic fitness	Team	Collisions	NR	NR	NR	34.5 (18.3)	NR	NR	NR	0.63 (0.15)
Hulin et al. [35]	Catapult (Optimeye S5)	NRL (NR)		Team	Collisions	NR	NR	NR	47.5 (10.5)	NR	NR	NR	NR
Hulin et al. [55]	Catapult (minimaxX S4)	NRL (2014)	Higher success	Hit-up forwards	Collisions	NR	NR	NR	NR	NR	NR	NR	0.84 (0.27)
				Adjustables	Collisions	NR	NR	NR	NR	NR	NR	NR	0.79 (0.25)
				Outside backs	Collisions	NR	NR	NR	NR	NR	NR	NR	0.36 (0.07)
			Lower success	Hit-up forwards	Collisions	NR	NR	NR	NR	NR	NR	NR	0.69 (0.19)
				Adjustables	Collisions	NR	NR	NR	NR	NR	NR	NR	0.45 (0.31)
				Outside backs	Collisions	NR	NR	NR	NR	NR	NR	NR	0.32 (0.12)
Johnston et al. [56]	Catapult (minimaxX S4)	International Student Competition (NR)		Team	Tackles	NR	NR	NR	18.96* (4.97)	NR	NR	NR	0.19* (0.03)
Kempton et al. [58]	GPSports (SPI-Pro)	NRL (2010–2011)		Team	Tackles	NR	NR	NR	(NR)	NR	NR	NR	0.6 (NR)
					Ball carries	NR	NR	NR	10.3 (NR)	NR	NR	NR	1.1 (NR)
Kempton et al. [42]	GPSports (SPI-Pro X)	NRL (2012)	Lower success	Team	Collisions	NR	NR	NR	22.2 (15.8)	NR	NR	NR	0.4 (0.5)
		NRL (2014)	Higher success	Team	Collisions	NR	NR	NR	18.6 (26.1)	NR	NR	NR	0.3 (0.6)
Murray et al. [61]	Catapult (minimaxX)	NRL (NR)	Shorter recovery	Team	Collisions	NR	NR	NR	43.0 (4.0)	NR	NR	NR	0.7 (0.1)
			Medium recovery	Team	Collisions	NR	NR	NR	35.0 (2.0)	NR	NR	NR	0.6 (0.1)
			Longer recovery	Team	Collisions	NR	NR	NR	38.0 (4.0)	NR	NR	NR	0.8 (0.1)
Varley et al .[16]	Catapult (minimaxX)	NRL (2009–2010)		Team	Collisions	0.3 (0.6)	7.5 (5.0)	11.1 (4.2)	18.9 (8.1)	0.00 (0.00)	0.13 (0.11)	0.19 (0.10)	0.33 (0.16)
Weaving et al. [7]	Catapult (Optimeye S5)	SL (2017)	1st half	Hit-up forwards	Collisions	NR	NR	NR	9.7 (2.6)	NR	NR	NR	NR
				Adjustables	Collisions	NR	NR	NR	5.5 (3.3)	NR	NR	NR	NR
				Outside backs	Collisions	NR	NR	NR	5.1 (2.6)	NR	NR	NR	NR
				Fullback	Collisions	NR	NR	NR	5.2 (3.0)	NR	NR	NR	NR
				Wide-running forwards	Collisions	NR	NR	NR	8.9 (2.7)	NR	NR	NR	NR
			2nd half	Hit-up forwards	Collisions	NR	NR	NR	9.1 (2.8)	NR	NR	NR	NR
				Adjustables	Collisions	NR	NR	NR	5.2 (3.2)	NR	NR	NR	NR
				Outside backs	Collisions	NR	NR	NR	4.9 (2.5)	NR	NR	NR	NR
				Fullbacks	Collisions	NR	NR	NR	5.7 (3.6)	NR	NR	NR	NR
				Wide-running forwards	Collisions	NR	NR	NR	8.2 (2.7)	NR	NR	NR	NR

*Collisions* collisions are reported but not differentiated into tackles and/or ball carries, *Impacts* impacts are reported based on *g* forces acting on the accelerometer, *NR* not reported, *NRL* National Rugby League, *NYC* National Youth Competition, *QCup* Queensland Cup, *SL* Super League, *Team* results for the entire team. *Data mean (±SD) were derived from individual games results

Microtechnology devices from three separate manufacturers were utilised across eight studies (see Table 4) with five different *g* force zone systems utilised by summating the tri-axial accelerometer force into zones 1 to 6. Of the 26 microtechnology studies, eight reported collisions according to intensity (i.e. mild, moderate or heavy) (see Table 5), whilst eight reported the absolute and relative force of collisions (as measured via *g* force) across individual thresholds (e.g. zones 1 through to 6) (see Tables 5 and 6). A number of these studies (*n* = 5) utilised an impact metric for these zones that encompasses all forces acting on the accelerometer including from actions other than collisions (see Table 6) [38, 59, 60, 67, 69].

**Table 5:** Zone characteristics for microtechnology devices using specific *g* force zones

Device	Studies	Zone 1	Zone 2	Zone 3	Zone 4	Zone 5	Zone 6
SPI-Pro X	Cummins and Orr [44]	< 5.9 *g*	6.0–6.9 *g*	7.0–7.9 *g*	8.0–9.9 *g*	10.0–11.9 *g*	> 12.0 *g*
SPI-Pro XII	Cummins and Orr [26]	< 5.9 *g*	6.0–6.9 *g*	7.0–7.9 *g*	8.0–9.9 *g*	10.0–11.9 *g*	> 12.0 *g*
SPI-Pro XII	Evans et al. [69]	7–9 *g*	9–11 *g*	11–13 *g*	13–15 *g*	NR	NR
SPI-Pro	Lovell et al [59]; McLellan and Lovell [60]; McLellan et al [38]; Weaving et al. [67].	5–6 *g*	6.01–6.5 *g*	6.51–7.0 *g*	7.01–8.0 *g*	8.01–10.0 *g*	> 10.0 *g*
MinimaxX S4	Oxendale et al [62].	2–3 *g*	3–4.5 *g*	4.5–6 *g*	6–8 *g*	> 8 g	NR

*NR* not reported

**Table 6:** Characteristics of collisions during match-play recorded by detection from microtechnology devices by studies reporting collision by intensity (g force) zones (Z1–Z6)

Study	Competition (season[s])	Microtechnology provider (device)	Groups	Positional group	Type of collision recorded	Frequency of collisions (*n*=) mean (±SD)							Relative frequency of collisions (*n min*^−1^*=*) mean (±SD)						
						Z1	Z2	Z3	Z4	Z5	Z6	Total	Z1	Z2	Z3	Z4	Z5	Z6	Total
Cummins and Orr [44]	NRL (2008)	GPSports (SPI-Pro X)		Team	Tackles	0 (NR)	0 (NR)	1 (NR)	1 (NR)	3 (NR)	1 (NR)	NR	NR	NR	NR	NR	NR	NR	NR
					Ball carries	0 (NR)	1 (NR)	0 (NR)	0 (NR)	2 (NR)	1 (NR)	NR	NR	NR	NR	NR	NR	NR	NR
Cummins and Orr [26]	NRL (2012)	GPSports (SPI-Pro X)		Hit-up forwards	Tackles	2.4 (NR)	1.7 (NR)	2.6 (NR)	7.2 (NR)	6.4 (NR)	1.3 (NR)	NR	0.10 (NR)	0 (NR)	0.10 (NR)	1.20 (NR)	0.20 (NR)	0.03 (NR)	NR
					Ball carries	0.2 (NR)	0.2 (NR)	0.2 (NR)	1.5 (NR)	4.6 (NR)	2.3 (NR)	NR	0 (NR)	0.01 (NR)	0 (NR)	0.04 (NR)	0.01 (NR)	0.01 (NR)	NR
					Tackle and ball carries	2.6 (NR)	1.9 (NR)	2.6 (NR)	8.7 (NR)	11 (NR)	3.5 (NR)	NR	0.10 (NR)	0.10 (NR)	0.10 (NR)	0.20 (NR)	0.30 (NR)	0.10 (NR)	NR
				Wide-running forwards	Tackles	1.8 (NR)	1.4 (NR)	2.4 (NR)	7.2 (NR)	5.6 (NR)	1.7 (NR)	NR	0.04 (NR)	0.02 (NR)	0.10 (NR)	1.20 (NR)	0.20 (NR)	0.03 (NR)	NR
					Ball carries	0.1 (NR)	0.1 (NR)	0.2 (NR)	1.9 (NR)	4.6 (NR)	1.6 (NR)	NR	0 (NR)	0 (NR)	0.01 (NR)	0.04 (NR)	0.10 (NR)	0.04 (NR)	NR
					Tackle and ball carries	1.8 (NR)	1.6 (NR)	2.7 (NR)	9.13 (NR)	10.33 (NR)	3.33 (NR)	NR	0.04 (NR)	0.03 (NR)	0.05 (NR)	0.17 (NR)	0.21 (NR)	0.07 (NR)	NR
				Adjustables	Tackles	1.1 (NR)	1.13 (NR)	1.9 (NR)	7.3 (NR)	4.5 (NR)	1.2 (NR)	NR	0 (NR)	0.02 (NR)	0.05 (NR)	0.20 (NR)	0.10 (NR)	0.04 (NR)	NR
					Ball carries	0.1 (NR)	0.1 (NR)	0.2 (NR)	1.5 (NR)	2.6 (NR)	0.6 (NR)	NR	0 (NR)	0 (NR)	0 (NR)	0.02 (NR)	0.05 (NR)	0.01 (NR)	NR
					Tackles and ball carries	1.3 (NR)	1.3 (NR)	2.1 (NR)	8.1 (NR)	7.2 (NR)	1.8 (NR)	NR	0.02 (NR)	0.02 (NR)	0.05 (NR)	0.19 (NR)	0.16 (NR)	0.04 (NR)	NR
				Outside backs	Tackles	1.2 (NR)	0.5 (NR)	1.1 (NR)	2.5 (NR)	1.5 (NR)	0.2 (NR)	NR	0.01 (NR)	0 (NR)	0.01 (NR)	0.03 (NR)	0.02 (NR)	0 (NR)	NR
					Ball carries	0.5 (NR)	0.2 (NR)	0.5 (NR)	2.8 (NR)	5 (NR)	2.2 (NR)	NR	0.01 (NR)	0 (NR)	0.01 (NR)	0.03 (NR)	5.40 (NR)	0.03 (NR)	NR
					Tackles and ball carries	1.7 (NR)	0.7 (NR)	1.6 (NR)	5.4 (NR)	6.5 (NR)	2.4 (NR)	NR	0.02 (NR)	0.01 (NR)	0.02 (NR)	0.06 (NR)	0.08 (NR)	0.03 (NR)	NR
Evans et al. [69]	SL (2012)	GPSports (SPI-Pro XII)		Hit-up forwards	Impacts	166 (70)	23 (8)	7 (2)	0.5 (0.2)	NR	NR	NR	3.29 (0.95)	0.47 (0.14)	0.15 (0.07)	0.01 (0.01)	NR	NR	NR
				Wide-running forwards	Impacts	177 (48)	24 (8)	6 (3)	0.6 (0.5)	NR	NR	NR	2.31 (0.49)	0.31 (0.07)	0.09 (0.04)	0.01 (0.01)	NR	NR	NR
				Adjustables	Impacts	200 (67)	18 (4)	5 (2)	0.3 (0.4)	NR	NR	NR	2.75 (0.79)	0.26 (0.07)	0.06 (0.03)	0.00 (0.01)	NR	NR	NR
				Outside backs	Impacts	135 (41)	17 (3)	6 (2)	0.7 (0.4)	NR	NR	NR	1.55 (0.47)	0.20 (0.03)	0.06 (0.02)	0.01 (0.00)	NR	NR	NR
Lovell et al. [59]	NRL (NR)	GPSports (SPI-Pro)		Team	Impacts	NR	NR	NR	NR	NR	NR	451. 0 (493.0)	NR	NR	NR	NR	NR	NR	13.00 (15.00)
McLellan and Lovell [60]	NRL (NR)	GPSports (SPI-Pro)		Team	Impacts	248.0 (140.0)	178.0 (93.0)	199.0 (138.0)	142.0 (81.0)	78.0 (48.0)	37.0 (18.0)	830. 0 (135.0)	NR	NR	NR	NR	NR	NR	NR
				Backs	Impacts	205.0 (106.0)	183.0 (65.0)	178.0 (49.0)	120.0 (57.0)	48.0 (36.0)	32.0 (5.0)	795.0 (145.0)	NR	NR	NR	NR	NR	NR	NR
				Forwards	Impacts	260.0 (43.0)	173.0 (98.0)	212.0 (101.0)	154.0 (44.0)	55.0 (17.0)	41.0 (22.0)	858.0 (125.0)	NR	NR	NR	NR	NR	NR	NR
McLellan et al. [38]	NRL (NR)	GPSports (SPI-Pro)		Team	Impacts	215.0 (110.0)	150.0 (90.0)	366.0 (172.0)	49.0 (28.0)	28.0 (14.0)	21.0 (8.0)	830.0 (135.0)	NR	NR	NR	NR	NR	NR	NR
				Backs	Impacts	214.0 (126.0)	154.0 (105.0)	334.0 (195.0)	50.0 (31.0)	26.0 (14.0)	20.0 (5.0)	795.0 (145.0)	NR	NR	NR	NR	NR	NR	NR
				Forwards	Impacts	215.0 (80.0)	146.0 (68.0)	392.0 (151.0)	47.0 (24.0)	29.0 (14.0)	21.0 (8.0)	858.0 (125.0)	NR	NR	NR	NR	NR	NR	NR
Oxendale et al. [62]	SL (2014)	Catapult *(minimaxX S4)*		Backs	Collisions	11.5 (7.1)	13.9 (4.5)	4.1 (3.0)	1.1 (1.4)	0.5 (0.7)	–	31.1 (13.1)	NR	NR	NR	NR	NR	NR	NR
				Forwards	Collisions	24.2 (16.0)	21.3 (13.2)	6.1 (6.0)	1.8 (2.7)	0.7 (1.9)	–	54.1 (37)	NR	NR	NR	NR	NR	NR	NR
Weaving et al. [67]	SL (2011–2013)	GPSports *(SPI-Pro XII)*	Training—small sided games	Team	Impacts	NR	NR	NR	NR	NR	NR	1835.0 (1819.0)	NR	NR	NR	NR	NR	NR	NR
			Training—skills	Team	Impacts	NR	NR	NR	NR	NR	NR	1069.0 (965.0)	NR	NR	NR	NR	NR	NR	NR
			Training—conditioning	Team	Impacts	NR	NR	NR	NR	NR	NR	3203.0 (2490.0)	NR	NR	NR	NR	NR	NR	NR
			Training—speed	Team	Impacts	NR	NR	NR	NR	NR	NR	603.0 (400.0)	NR	NR	NR	NR	NR	NR	NR
			Training—strongman	Team	Impacts	NR	NR	NR	NR	NR	NR	391.0 (428.0)	NR	NR	NR	NR	NR	NR	NR
			Training—wrestle	Team	Impacts	NR	NR	NR	NR	NR	NR	269.0 (261.0)	NR	NR	NR	NR	NR	NR	NR

*Collisions* collisions are reported but not differentiated into tackles and/or ball carries, *Impacts* impacts are reported based on g forces acting on the accelerometer, *NR* not reported, *NRL* National Rugby League, *SL* Super League, *Team* results for the entire team

Individual studies reported differences in collisions between levels of higher and lower aerobic fitness (estimated VO_2_ max) [53], differences between the first half and second half [7], and differences in collisions over a season between short, medium, and longer turn-around times [61]. One study investigated collisions during training modes over a season [24], one study investigated differences in collisions from total match-time and normalised to ball-in-play time [48], and two studies reported differences between successful and less-successful teams [42, 54].

### Collision Frequency

#### Video Notational Analysis

**Fig. 2:**
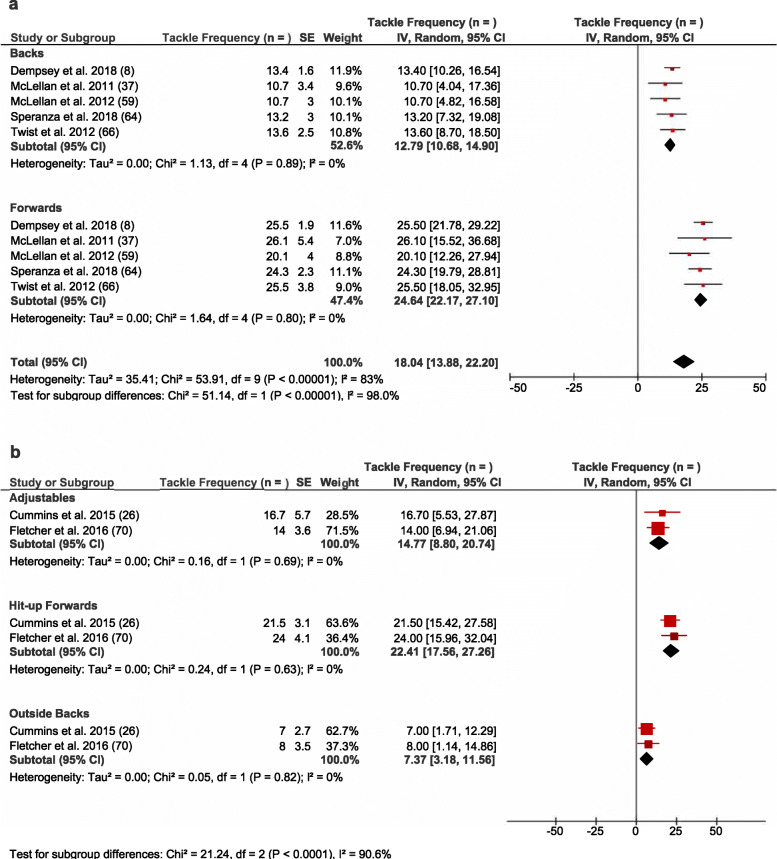
Meta-analysis of studies reporting absolute tackle frequency (*n*) from video analysis. The forest plot (mean and 95% confidence interval [CI]) was used to present the results of the meta-analysis and combined pooled estimates for absolute tackle frequency for **a** backs and forwards and **b** at the positional group level. Within the plot, squares and horizontal lines represent individual study mean and 95% CI and diamonds represent pooled mean and 95% CI

**Fig. 3:**
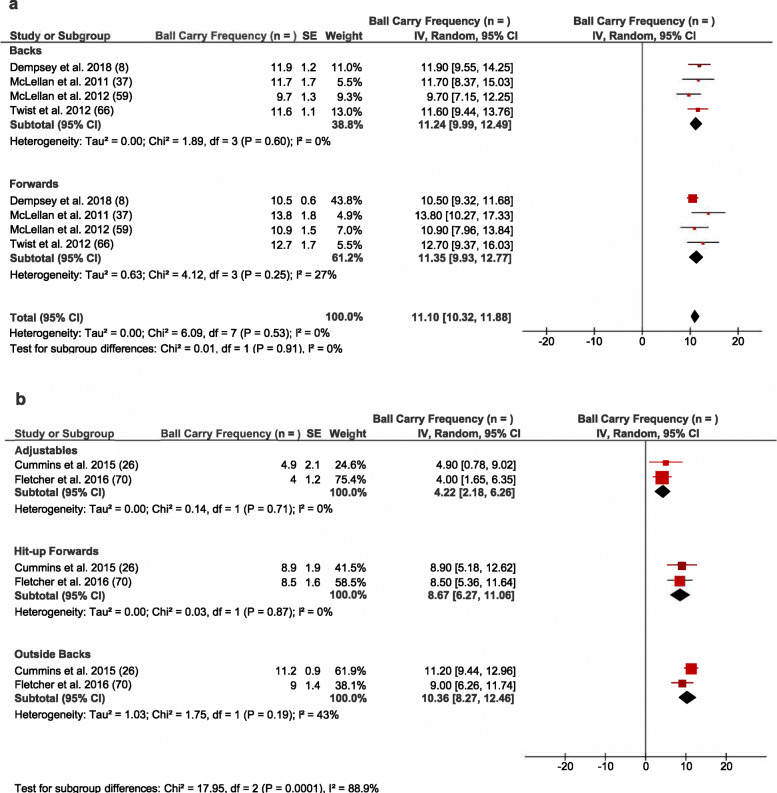
Meta-analysis of studies reporting ball carry frequency (*n*) from video analysis. This forest plot (mean and 95% confidence interval [CI]) was used to present the results of the meta-analysis and combined pooled estimates for absolute ball carry frequency for **a** backs and forwards and **b** at the positional group level. Within the plot, squares and horizontal lines represent individual study mean and 95% CI and diamonds represent pooled mean and 95% CI

Overall, 11 studies reported on absolute collision frequency per match using video notational analysis at either the team or positional group level (see Table 3). A pooled analysis of these studies identified that forwards completed approximately twice the number of tackles per game than backs (*n* = 24.6 vs 12.8 per match, *I*^*2*^ = 83%) (see Fig. 2a), whilst the average number of ball carries remained relatively similar between forwards and backs (*n* = 11.2 vs 11.4 per match, *I*^2^ = 0%) (see Fig. 3a). There were positional subgroup differences (*I*^*2*^ = 87.6%; see Fig. 2b), with the hit-up forwards (*n* = 22.4) undertaking a greater number of tackles per match than the adjustables (*n* = 14.7) and outside backs (*n* = 7.4). Heterogeneity within positional groups was low for adjustables, hit-up forwards, and outside backs (*I*^2^ < 25%). Two studies investigated tackles at the team level and reported 14.9–19.9 tackles per match [37, 60]. Two studies found wide-running forwards complete a similar number of tackles per match as hit-up forwards [26, 49], but the data integrity meant they could not be included in the subgroup analysis.

Conversely, both backs and forwards completed similar ball carries per match (see Fig. 3a). This result was mirrored when examining the positional subgroups with outside backs (*n* = 10.4), and hit-up forwards (*n* = 8.7) completing a somewhat similar number of ball carries per match (see Fig. 3b). However, adjustables (*n* = 4.2) undertook considerably fewer ball carries per match than all other positional groups (see Fig. 3b). Low heterogeneity was observed between studies which examined ball carry frequency in backs and forwards (*I*^2^ < 25%) (see Fig. 3a), with high heterogeneity (*I*^2^ > 75%) observed between studies reporting ball carries in each of the individual subgroups (see Fig. 3b). Three studies investigated ball carries across the team and observed that athletes complete 8.8–12.2 ball carries per match [37, 50, 60]. Two studies found wide-running forwards complete an average frequency of ball carries per match which is similar to outside backs (*n* = 7.9–17.0) [26, 49]; however, due to limited data, these results were not included in the pooled analysis.

**Fig. 4:**
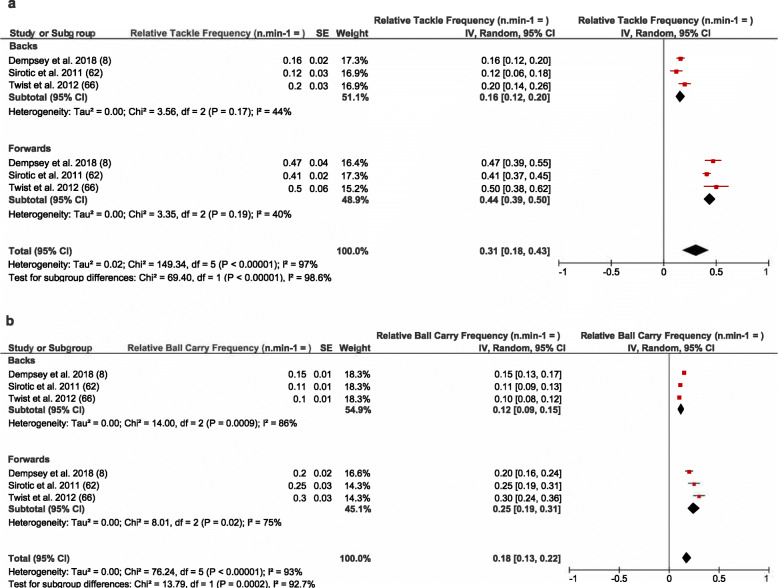
Meta-analysis of studies reporting relative (*n* min^−*1*^) tackle and ball carry frequency from video analysis. The forest plot (mean and 95% confidence interval [CI]) was used to present the results of the meta-analysis and combined pooled estimates for **a** relative tackle frequency for backs and forwards and **b** for relative ball carry frequency for backs and forwards. Within the plot, squares and horizontal lines represent individual study mean and 95% CI and diamonds represent pooled mean and 95% CI

Of the included studies, six reported relative collision frequency per match utilising video notational analysis at either the team and or positional group level. Pooled analysis of these studies identified that forwards undertook a greater relative frequency of tackles per match when compared to backs (*n* min^−1^ = 0.44 vs. 0.16) (see Fig. 4a). In one study, there was a higher frequency of collisions reported for forwards during defensive (*n* min^−1^ = 1.9) as opposed to offensive (*n* min^−1^ = 0.8) phases of play [51]. On the other hand, ball carry frequency relative to playing time was higher in the forwards when compared to backs (*n* min^−1^ = 0.25 vs. 0.11) (see Fig. 4b). Moderate heterogeneity was observed within studies reporting relative tackle frequency in both backs and forwards (*I*^2^ = 25–49%) (see Fig. 4a). Conversely, studies which examined relative ball carry frequency reported high heterogeneity in forwards and backs (*I*^2^ = ≤ 60%) (see Fig. 4b). Pooled analysis at the positional group level was not undertaken due to the limited number of studies (*n* = 4 studies [Table 3]).

Four studies reported on absolute collision frequency at the game or competition level via video notational analysis [1, 28, 41, 68] (see Table 3). At the competition level, there were differences between the frequency of collisions undertaken within NRL and SL matches, with SL teams completing more ball carries per match, with a relatively similar tackle load [1]. Furthermore, Woods et al. identified that NRL teams complete a greater number of tackles and ball carries when compared to their NYC counterparts [68]. Finally, King et al. observed similar overall collision demands for both tackles and ball carries between international matches played at the Rugby League World Cup, and NRL competition standard [41]. However, there were differences at the positional group level with a forwards and backs undertaking a greater number of tackles and ball carries respectively, at both the international and NRL levels [41].

#### Microtechnology

**Fig. 5:**
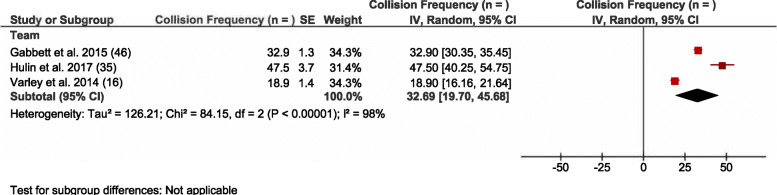
Meta-analysis of studies reporting collision frequency (*n*) derived from microtechnology. The forest plot (mean and 95% confidence interval [CI]) was used to present the results of the meta-analysis and combined pooled estimates for absolute collision frequency at the team level. Within the plot, squares and horizontal lines represent individual study mean and 95% CI and diamonds represent pooled mean and 95% CI

Collectively, 18 studies reported comparisons that utilised microtechnology to assess absolute or relative collision frequency, and collision intensity through descriptor zones (i.e. mild, moderate or heavy) or *g* force intensity zones (see Table 5). Eleven of these studies reported absolute collision frequency (*n=*) with 31 different comparison cohorts extracted from these studies. Pooled analysis of these comparisons identified microtechnology-based studies reporting 32.7 collisions per match from the three studies reporting collisions at the team level (see Fig. 5). Studies that reported collision frequency from microtechnology at the team level exhibited high heterogeneity (*I*^2^ = 98%) (see Fig. 5).

Studies using microtechnology reported that forwards undertook between 18.3 and 44.0 collisions per match on average, whilst one study reported that backs undertook 26.0 collisions per match (see Table 4) [23, 47, 52]. When examined at the positional group level, adjustables completed 16.4 to 34.0 collisions per match, whilst studies reported that outside backs complete between 14.8 and 28.0 collisions per match [23, 24, 47]. Only one study reported collisions per match for hit-up forwards and wide-running forwards based on microtechnology [31], with hit-up and wide-running forwards undertaking 37.0–42.0 and 28.0–45.0 collisions per match, respectively [24, 31]. Due to insufficient datasets (i.e. less than two studies), further analysis was not undertaken on this data.

Gabbett et al. investigated the collision demands of training and competition and reported similar collision demands between skill focused training and repeated high-intensity effort training sessions [24]. Each training modality reportedly involved lower absolute collision demands at both the team and individual positional group when compared to match-play. However, on a relative basis, the collision demands of training were similar to match-play for both repeated high-intensity effort and game-based training modes (see Table 4). Furthermore, Gabbett et al. identified that athletes with a higher predicted aerobic capacity (i.e. VO_2_max) demonstrate an increased absolute collision frequency [53].

Research has investigated collision demands for athletes competing on teams that finished the 2012 and 2014 NRL seasons with greater, or lesser, success based on final ladder position [42]. The collision demands of more successful teams were reported to be greater than those associated with lower success on both an absolute and relative basis. A shorter (5–6 days) turn-around between matches was linked with a greater collision when demand compared to medium (7–8 days) or longer turn-around (> 9 days) [61]. Finally, the collision demands of the first half of matches were similar to those of the second half across all positional subgroups [7].

For relative collision frequency as reported via microtechnology, forwards completed between 0.35 and 0.77 *n* min^−1^ of match-play [23, 47, 52], which was greater than the one study which reported the relative frequency of backs (*n* min^−1^ = 0.30) [52]. These differences were mirrored when relative collisions were examined at the positional subgroup level, with hit-up forwards undertaking greater collisions per minute (*n* min^−1^ = 0.61–1.09) than adjustables (*n* min^−1^ = 0.29–0.58) and outside backs (*n* min^−1^ = 0.19–0.36) (see Table 4) [23, 24, 47]. Only one study investigated the relative frequency of collisions completed by wide-running forwards as reported via microtechnology, identifying that wide-running forwards undertook 0.76 collisions per min of match-play [31]. Furthermore, there was a higher frequency of collisions reported per minute of match-play for forwards during defensive phases of play (*n* min^−1^ = 1.9) than during offensive phases (*n* min^−1^ = 0.8) [51]. These data were not entered into the meta-analysis due to insufficient data.

### Collision Intensity

#### Video Notational Analysis

Three studies reported metrics associated with the intensity of collisions as subjectively derived from video analysis [31, 33, 71]. One study examined the velocity (m s^−1^) into contact between rugby league athletes competing at different competition levels, concluding that both professional and semi-professional athletes undertake a similar velocity into contact (2.91 vs. 2.76 m s^−1^) [71]. Another study investigated the influence of progressive fatigue on acceleration into contact through frame by frame analysis of video and reported a gradual decrease in acceleration with increased fatigue [33]. More specifically, acceleration was reported to be 3.8 m s−^2^ with low fatigue, 2.3 m s^−2^ during moderate fatigue, and 1.7 m s^−2^ during periods of heavy fatigue.

Finally, one study investigated collision intensity by characterising each collision through a mild, moderate, or heavy rating system [31]. In this system, a mild collision occurred when a player made contact with a player but was able to continue forward progress, and a moderate collision was coded when an athlete made contact and momentum continued until finally being tackled. Lastly, a heavy collision was coded when momentum was halted and the athlete forced backwards [31]. Of the 237 collisions analysed using this system, 24 were considered mild, 46 were considered moderate, and 119 were considered heavy. This represented a 63% difference between mild and moderate collisions, and a 133% difference between mild and heavy coded collisions. Following coding of the collisions via video, the system was then compared to synched microtechnology-derived collision frequencies in each intensity zone [31].

#### Microtechnology

**Fig. 6:**
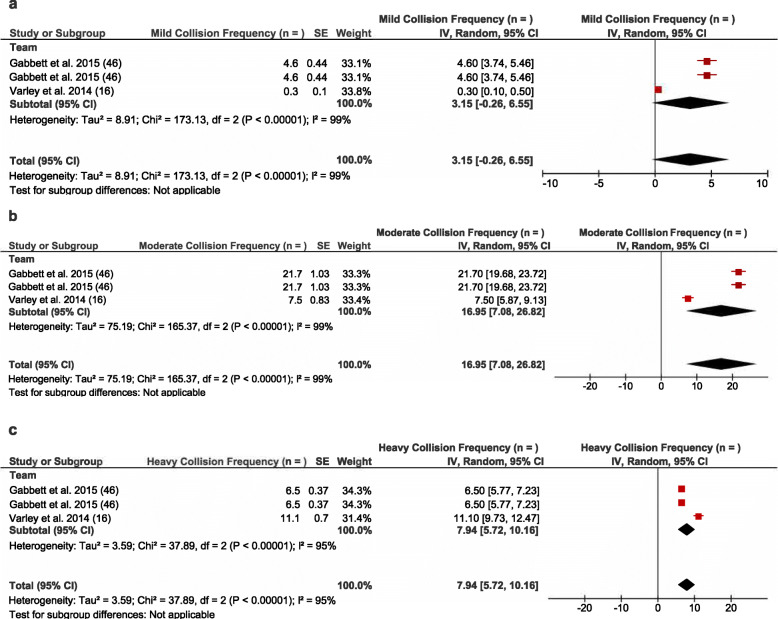
Meta-analysis of studies reporting mild/moderate/heavy collision frequency (*n*) at the team level from microtechnology. The forest plot (mean and 95% confidence interval [CI]) was used present the results of the meta-analysis and combined pooled estimates of team collision frequency for **a** mild, **b** moderate, and **c** heavy collisions. Within the plot, squares and horizontal lines represent individual study mean and 95% CI and diamonds represent pooled mean and 95% CI

Six studies [16, 23, 24, 31, 48, 51] reported collision intensity based on mild, moderate, and heavy collisions which were reportedly derived from microtechnology. Pooled analysis of these studies that reported similar positional groupings identified that on average there were 3.2 mild collisions per match at the team level (see Fig. 6a). A larger frequency of moderate (*n* = 17.0 per match) (see Fig. 6b) and heavy collisions (*n* = 7.9 per match) (see Fig. 6c) were observed from the pooled analysis of collisions per match. High heterogeneity was observed within studies that reported mild, moderate, and heavy collisions (*I*^2^ > 75%) (see Fig. 6).

At the positional group level, hit-up (*n* = 2.0–4.0) and wide-running forwards (*n* = 2.0–4.0) were associated with the greatest number of mild collisions per match when compared to outside backs (*n* = 0.2–5.0) and adjustables (*n* = 0.3–4.0) [23, 24, 49]. These positional group differences were mirrored within moderate collisions with wide-running (*n* = 12–24) and hit-up forwards (*n* = 20.0–22.0) completing more moderate collisions than adjustables (*n* = 6.5–19.0) and outside backs (*n* = 4.3–12.0) [23, 24, 49]. Finally, hit-up (*n* = 15.0–16.0) and wide-running forwards (*n* = 15.0–17.0) completed a greater frequency of heavy collisions per match than outside backs (*n* = 2.0–12.0) and adjustables (*n* = 9.4–15.0) [23, 24, 49]. The greatest frequency of collisions for each of the positional groups fell into the moderate collision category. There were limited data across all positions at the subgroup level to enter the studies into the meta-analysis.

Eight of the included studies [26, 38, 44, 59, 60, 62, 67, 69] reported collision intensity based on accelerometer load (*g* forces) which was divided into discrete intensity zones (see Table 5). These studies reported on five unique intensity zones ranging from 2–3 *g* to 13–16 *g* zone configurations from four microtechnology devices manufactured by GPSports (SPI-Pro, SPI-Pro X, SPI-Pro XII devices) and Catapult (minimaxX S4 device) (see Table 5).

A number of these studies [38, 59, 60, 69] reported collisions using a manufacturer-derived impact metric (see Table 6). This metric included forces acting on the accelerometer from all actions including tackles, foot strikes, and rapid accelerations which are dependent on the mass and movements of the individual athlete [4]. Due to this, these studies reported a frequency of total collisions (~ 800 per match) (see Table 6) which is far in excess of other research reporting collisions using microtechnology (*n* = 16.0–37.0) [24, 31], or from video analysis of tackles (*n* = 14.9 ± 10.5) and hit-ups (*n* = 10.2 ± 3.8) [38]. Similarly, this frequency of collisions is significantly greater than the absolute collision frequency derived from microtechnology in the current meta-analysis (*n* = 32.7). Given the disparity between reporting impacts and collisions, and in the zone classification systems utilised in these studies, further comparisons were not able to be performed as the methods and data were heterogeneous.

## Discussion

To our knowledge, this is the first systematic review, including a meta-analysis to specifically synthesise the current methods of analysing the frequency and intensity of collisions in the rugby league. The review clearly demonstrates that video-based notational analysis and microtechnology devices are the two primary methods utilised to examine the frequency and intensity of collisions in the rugby league. Collectively, forwards experience a greater dose of collisions than backs which is primarily attributable to a greater tackle frequency, with ball carry frequency demands slightly higher for forwards compared to backs. Overall, collisions have been quantified using a variety of data collection methods with a lack of consistency in regards to positional groups as well as intensity descriptors. Finally, there has been a lack of validation research for the use of microtechnology in assessing collision frequency and intensity. Therefore, practitioners should explore independently validating microtechnology in their context to ensure they are appropriately monitoring collision loads.

### Collision Frequency

From a video analysis perspective, there are disparities in the collision demands across positional groups, with forwards completing a greater number of collisions per match when compared to backs. This disparity is primarily associated with a greater tackle frequency for forwards, with ball carry frequency being more similar across the positional groups. These tackle and ball carries reflect the relative demands of match-play, with forwards exhibiting a near threefold increase in the number of tackles per minute of match-play when compared to backs, with the relative number of ball carries being higher for forwards and their positional subgroups. This pattern likely reflects the tactical demands of the modern rugby league, with teams utilising interchange players to complete tackles and ball carries during their time on field. This translates to a greater workload for these players who occupy the middle of the defensive line (i.e. the forward positional groups), resulting in a greater number of collisions completed relative to the players’ time on field.

These findings were also reflected in the absolute and relative collision frequencies as quantified via microtechnology. Forwards and their positional subgroups of hit-up and wide-running forwards complete a greater number of collisions in absolute terms and relative to their time on the field of play. High heterogeneity was observed in the collision demands derived from microtechnology at the team and positional subgroup level. This may be due to the majority of studies focusing on a single team or cohort, and the unique physical and tactical aspects placed upon those players not generalising across teams or cohorts. Alternatively, the high heterogeneity might be a function of the different microtechnology devices utilised between studies. Further granular analysis of differences in the type of collisions is presently not possible, as current microtechnology does not permit the differentiation of collisions into tackles and ball carries.

The lack of consensus regarding the definition of tackles and collisions is a potential issue within the current literature. Specifically, a number of video-based studies used the definition described by Gissane et al. [72] wherein a tackle is defined as when ‘… the ball carrier is held by one or more of the opposing players and either the ball or hand of the arm holding the ball makes contact with the ground or the ball carriers cannot make further progress.’ [28, 41, 49]. Other studies focusing on microtechnology-based collisions, however, have defined the collision as occurring when a player makes contact with another player or the ground, which results in an alteration to the player’s momentum or direction of travel [31, 35]. The differences in definitions of tackles and collisions have the potential to alter the frequency in which these actions are counted and hinder the ability to translate findings into practical recommendations.

### Collision Intensity

Compared to literature quantifying the absolute and relative collision frequency demands of match-play, there is a paucity of research on the intensity of collisions within the rugby league. Further limiting such research is the lack of consistency in the quantification of collision intensity between studies. Through video analysis, a number of studies have attempted to quantify collision intensity by calculating the velocity and accelerations into contact [71, 73]. This process involves manually coding video frame-by-frame before the athlete enters a collision to derive velocity and acceleration and as such is time-consuming [73] and may be influenced by the subjective nature of video analysis. The time taken to code each frame and the manual nature of this process means that this information cannot be used in real time or close to real time to influence decision making. Using velocity into contact as an intensity metric makes a number of assumptions that primarily relate to velocity equating to impact forces, and in turn into dominance in the collision. This relationship has been examined in the rugby union, whereby velocity into contact, but not impact force, was greater for dominant tackles and carries when compared to neutral and passive tackles and ball carries [74]. Gabbett and Ryan investigated the differences between professional and semi-professional rugby league athletes in regard to velocity into contact and subjective ratings of tackle performance [71]. Despite large differences in tackle performance between competition standards, minimal differences were noted for velocity into contact, suggesting that collision dominance may not be related to the velocity that a player approaches the collision. To our knowledge, as this is the only study to investigate velocities and collision dominance in the rugby league, further research in this area is warranted.

Literature examining collision intensity via microtechnology has also been limited due to methodological inconsistencies with accelerometer-derived classification zones used to categorise impacts from low- to high-intensity zones [60]. In theory, this process provides practitioners with an understanding of the accelerometer load that includes the accumulation of the tri-axial forces acting upon the accelerometer from actions such as change of direction movements, accelerations and decelerations, and collisions [59]. As such, an impact metric provides limited information for the practitioner in regard to collision intensity and instead is indicative of a ‘global’ accelerometer load [4]. Furthermore, intensity-based research has employed a number of different devices with four to six different intensity zone categories ranging from 2–3 *g* to > 12 *g* (see Table 5) and estimated collision intensity via qualitatively grouping collisions into categories of mild, moderate, or heavy. Given the lack of methodological consistency within and between studies utilising *g* force-based intensity zones, further comparison and analysis of these findings is difficult. As such, as previously highlighted by Cummins et al. [14], a consensus on the definition of zone intensities and descriptors for both impact metrics and accelerometer loads is required in order to facilitate comparisons within individual sporting codes and levels of participation. Such consistency could provide insights which are generalisable between teams and positional groups, enabling meaningful analyses, and ensuring athletes are exposed to appropriate collision loads, which is critical to both injury-prevention and physical conditioning.

Beyond the initial velocity and contact force, another factor that may influence the intensity of the collision is the post-contact wrestle phase of the tackle or ball carry. During this phase, the athletes involved wrestle or grapple to achieve dominance in the tackle and this necessitates large muscular force generation whilst in a near stationary position [75]. These static exertions involve isometric and eccentric muscle actions that are likely to produce extensive muscle damage [27] and incur substantial energetic costs [75]. Despite the apparent high energetic demand of such events, microtechnology is incapable of quantifying the work completed in a stationary position due to minimal displacement of the device [75, 76]. As such, microtechnology devices are unable to account for the physiological demand that occurs during static exertions and are therefore not an appropriate tool to monitor collision intensity in the post-contact wrestle phase.

### Microtechnology Validity

There has been an increase in the number of studies examining and reporting the collision demands of rugby league training and match-play. The use of microtechnology to automatically quantify the frequency of collision events appears to be an emerging area of research. Despite this, there is minimal research into the validity of automated tackle detection algorithms and the utility of such devices in quantifying the frequency of collisions in the rugby league [35, 37].

To our knowledge, only two microtechnology devices from one manufacturer (i.e. Catapult minimaxX and Optimeye devices) have been validated for automated collision detection in the rugby league [35, 37]. The respective devices utilise an algorithmic approach to detect collisions via spikes in instantaneous PlayerLoad (arbitrary units [AU]) and changes in unit orientation that are detected via the gyroscope and magnetometer [31, 35, 37]. The Catapult minimaxX device has been utilised extensively to quantify collisions, with the 2010 validation study in rugby league training receiving over 100 citations to date [31]. Indeed, the authors of this study cite a near perfect correlation between microtechnology detected and video-coded collisions (*r* = 0.96) as evidence for the device’s automatic collision detection validity [31, 37]. Unfortunately, this approach to validation is potentially problematic as it fails to report a number of factors regarding the precision of this microtechnology for the detection of collision events. More specifically, the sensitivity, specificity, and accuracy of the device to identify collisions within rugby league match-play have not been elucidated [35]. Indeed, whilst there is a strong overall relationship between video-coded and microtechnology-derived collisions, large discrepancies in the relationship are observed, particularly with players who undertake fewer collisions (see Fig. 1 in [31]). Additionally, this research did not report the post-collection data processing that was undertaken, which is important for its reproducibility and usability. Collectively, this suggests that this device has yet to be appropriately validated for automated collision detection. As such, until the minimaxX device has been appropriately validated, practitioners should exercise caution when utilising this device in isolation (i.e. without video analysis of collisions) to quantify the frequency of collisions in field-based team sports.

The Catapult Optimeye S5 device has recently undergone validation for the collision detection algorithm utilising a criterion validity framework. Hulin et al. [35] compared microtechnology-detected collisions to video-coded collisions as a criterion measure during rugby league match-play. In this context, the true-positive was reported when a player was involved in a collision and the device recorded that collision, whilst a false-positive, was reported when the player was *not* involved in a collision and the microtechnology device recorded a collision. Conversely, a false-negative was reported when the player was involved in a collision and the device did not record the collision, and a true-negative was reported when (1) the microtechnology device recorded a > 2 AU PlayerLoad spike, (2) the player was *not* involved in a collision, and (3) the microtechnology device *did not* report a collision [35]. Following removal of short duration (< 2 s) and low-intensity (< 1 PlayerLoad AU) events, it was reported that the ability of the device to not report collision events when they do not occur (i.e. specificity) was 91.7%, and the ability to detect a collision when it did occur (i.e. sensitivity) was 97.6% [35]. Similarly, accuracy improved to 92.7% following the removal of short duration and low-intensity events during data processing, with the majority of false positives identified as being related to rapid change of directions. Whilst post-collection processing of the data to remove errors may be considered a limitation, this information enables applied practitioners to attain a similar level of accuracy when monitoring collisions via microtechnology. Indeed collisions of low duration and intensity may not be as physically or perceptually fatiguing as those of higher durations and intensities [27]. As such, it may be less pertinent to consider these collisions in the context of contact load monitoring. Given the limited validation research of collision frequency detection, further research investigating the validity of commercially available devices to quantify collision events is warranted [4].

Research that has attempted to explore collision intensity via microtechnology exhibits similar limitations. Specifically, in the original work describing subjective intensity classification [31], the authors fail to outline how the device is able to automate the categorisation of collisions based on subjective intensity (i.e. mild, moderate, heavy). From this study [31], it appears that the respective microtechnology device may not have the capacity to automate collision load monitoring, as the resulting analysis only identifies that the frequency of collisions is highly correlated to the number of collisions in each qualitative intensity descriptor (*r* = 0.89, 0.97, and 0.99 for mild, moderate and heavy collisions, respectively). As outlined previously, this approach does not identify the number of collisions that were or were not classified correctly using appropriate validity statistics (i.e. specificity, sensitivity, accuracy). A later study included microtechnology-based *g* forces alongside the qualitative descriptors of mild (1–2 *g*), moderate (2.1–4 *g*), and heavy (> 4 *g*) [31, 51]. Whether these *g* forces and associated zones represent the same zones as the original research is, however, unclear. Furthermore, the *g* forces associated with these zones are notably lower than the highest *g* force zones reported by other microtechnology devices and studies (see Table 5). This discrepancy highlights differences in microtechnology devices both within and between manufacturers with respect to hardware (i.e. inertial sensors, sampling frequency of both GPS and inertial sensors, and dissimilar chipset configurations) [9]. Given these disparities, it is not possible to generalise the validity or reliability-based findings of one device to that of another. With this in mind, any device or algorithm that is launched commercially needs to be appropriately validated against criterion measures, even if they are considered to be iterations of currently available and validated hardware, software, or algorithms (i.e. Catapult minimaxX and Optimeye devices). Until devices and algorithms have undergone appropriate external validation, practitioners and researchers alike should be cautious in interpreting the reported collision frequency or intensity information. This knowledge is important for practitioners to be cognisant of when selecting and using microtechnology.

Despite representing a relatively small portion of overall match-play time, collisions are one of the most physically demanding aspects of the rugby league [26]. With the increasing availability of microtechnology devices and their proliferation in the rugby league, both research and practice has shifted to an increasing reliance upon these devices to quantify every aspect of training and competition [4, 25, 77]. This shift has led to further innovation and automation in the approaches utilised to quantify collisions. Recently, a novel metric that purports to combine accelerometer impact forces, velocity into contact, and the collision duration has been developed by STATSports (STATSports, Northern Ireland) [74]. Developed utilising data from the rugby union, this collision load metric is described as a ‘… weighted score providing an intensity of each collision…’ [74]. Beyond this, specific information in regard to the algorithm and validation of the metric is currently lacking outside of the rugby union [74]. One challenge that remains in the validation of microtechnology to appropriately quantify collision intensity is the lack of an appropriate criterion to validate device metrics against. Whilst microtechnology collision frequency can be compared to video-based methods to establish criterion validity, currently no criterion measure exists in order to validate collision intensity. Indeed, various methods to quantify physical or collision workload intensity have been suggested in the research including subjective measures such as rating of perceived challenge [78], rating of perceived exertion (RPE) [79], and rating of mental effort [79]. Others have investigated intensity based on objective markers including muscle damage biomarkers (such as creatine kinase) [27] and shoulder impact forces [80]. As there is currently no gold standard and a lack of consensus in regard to the measure(s) that appropriately capture collision intensity, future research into the validation of collision intensity and load metrics as reported via microtechnology is warranted. Such metrics may provide further insight into the monitoring of training and game loads, injury prevention, and physical conditioning [4]. Despite this, no microtechnology device to date has the ability to differentiate between collisions, tackles and ball carries, or other sports-specific actions (missed tackles, scoring a try, offloads etc.) which involve collisions in the rugby league [4]. In other sports, recent research has shown the promise of machine learning methods to automate collision event detection [34, 81, 82]. This research indicated that utilising microtechnology data in the rugby union, machine learning approaches such as random forests and decision trees can accurately detect and quantify sports-specific actions, such as scrummaging [81], one-on-one tackling, and rucks [34]. It is clear that these approaches can distinguish between different features in large and complex, noisy datasets such as those regularly recorded from microtechnology in sport. Given the potential of such data processing methods, the application of machine learning methods to microtechnology data from the rugby league may enable the ability to differentiate collision events (e.g. tackles and ball carries).

### Limitations

A limitation of existing literature is that studies have utilised dissimilar approaches to grouping individual positions to various groups and subgroups. This has meant that there is a general lack of consistency across studies, with a number of studies utilising positional groupings that have not been replicated by later research. This lack of consistency may have contributed to the high heterogeneity that was present in the collision demands derived from microtechnology in the various groups and subgroups. Similarly, there is a lack of consistency regarding approaches to quantifying collision intensity from microtechnology. Studies have utilised a variety of different *g* force zones or subjective intensity descriptors that differ both within and between devices and manufacturers (see Tables 5 and 6). Furthermore, a number of studies in the review did not report the number of participants in each cohort or the number of microtechnology files that precluded their inclusion in the current meta-analysis. As such, as has previously been suggested [14], a consensus on the definition of positional groups, zone intensities, and descriptors for both impact metrics and accelerometer loads is required in order to facilitate comparisons within individual sporting codes and levels of participation.

### Future Directions

Although microtechnology has been comprehensively adopted in the male senior rugby league, independent validation of microtechnology in the detection of collisions is needed, as a number of current devices and algorithms have not been sufficiently externally validated. This is potentially problematic as practitioners may be using these devices with misplaced confidence in their ability to automate collision detection. Similarly, validation of microtechnology collision load metrics and their constituents against the physical force of collisions is warranted, as such information may be of interest to practitioners. Indeed, a study by Usman et al. [80] has investigated the forces of tackles in rugby union athletes using a static instrumented tackle bag. Peak impact force progressively declined with increasing levels of fatigue, and lower forces were observed in the non-dominant shoulder when compared to the dominant shoulder [80]. Further research that investigates aspects of the collision in dynamic situations has just begun to emerge in the wider research [83], and these models hold promise for the investigation of microtechnology collision intensity validation.

If researchers and practitioners are to continue to glean information from microtechnology devices using *g* force intensity zones, or qualitative descriptors, then they must be aware of the limitations of these approaches as highlighted within this review. Similarly, they should understand that due to inherent differences in the devices and associated algorithms, they are unable to generalise their data across different microtechnology devices and manufacturers. Each new device and detection algorithm that enters the market will need to be validated against criterion measures. This applies to iterations of previously validated devices.

Currently, microtechnology cannot appropriately quantify the post-contact wrestle phase of the collision, which is considered a highly fatiguing aspect of match-play [26]. To address this limitation, a move to utilising sophisticated analytic methods (such as machine learning) and a mechanical model to quantify these actions through the work-energy theorem have recently been suggested [75]. By applying these methods to microtechnology data, the relative contribution of locomotor and collision loads may be able to be partitioned and approximated appropriately. Whilst such approaches hold promise to collision modelling, they have yet to be fully elucidated.

Machine learning approaches have recently been utilised in a range of other sports for their ability to differentiate sports-specific actions in complex and noisy microtechnology datasets [34, 81, 84]. Investigating whether machine learning methods can differentiate between collision-based events such as tackles and ball carries from the overall collision pool is warranted. Automating this process would streamline analysis and provide practitioners with further detailed information on contact loads. This would inform short- and long-term collision load monitoring and allow for the exploration of interactions with contact-related injuries [31], contact adaptation [27], and the effects of contact skill and conditioning programmes [4, 85].

## Conclusions

The quantification of collisions has transitioned from video notational analysis methods to the use of microtechnology devices and associated algorithms to quantify both collision frequency and intensity. Differential collision profiles have been observed in the literature between forward and back positional groups and their distinct subgroups. The hit-up and tackle demands of forwards and backs differ, with forwards experiencing an increased absolute and relative frequency of tackles and collisions. Microtechnology has been utilised to quantify collision frequency and intensity, but a number of disparate approaches have been undertaken with little consensus to an ideal approach having been established. Furthermore, despite widespread popularity, a number of the microtechnology devices have not been appropriately validated for use in the rugby league. Future research using microtechnology should establish the criterion validity of current and novel devices with collision detection algorithms in measuring collision frequency. Similarly, future research should look to establish the measures that capture the intensity of collisions and examine the relationship between collision intensity metrics and directly assessed impact forces. Examining whether machine learning approaches can differentiate between collision-based events such as tackle and ball carry actions is warranted.

## Data Availability

Not applicable

## Abbreviations

CI: Confidence interval
GPS: Global Positioning System
Hz: Sampling frequency
NR: Not reported
NRL: National Rugby League
NSWCup: New South Wales Cup Competition
NYC: Australian National Under 20’s Youth Competition
QCup: Queensland Cup Competition
SL: Super League
