# Correction to: Quantifying the Collision Dose in Rugby League: A Systematic Review, Meta-analysis, and Critical Analysis

**DOI:** 10.1186/s40798-020-00263-w

**Published:** 2020-10-27

**Authors:** Mitchell Naughton, Ben Jones, Sharief Hendricks, Doug King, Aron Murphy, Cloe Cummins

**Affiliations:** 1 School of Science and Technology, University of New England, Armidale, NSW Australia; 2 Carnegie Applied Rugby Research (CARR) centre, Institute for Sport Physical Activity and Leisure, Leeds Beckett University, Leeds, UK; 3 Leeds Rhinos Rugby League club, Leeds, UK; 4 England Performance Unit, The Rugby Football League, Leeds, UK; 5 Division of Exercise Science and Sports Medicine, Department of Human Biology, Faculty of Health Sciences, University of Cape Town, Cape Town, South Africa; 6 Health through Physical Activity, Lifestyle and Sport Research Centre (HPALS), Faculty of Health Sciences, The University of Cape Town, Cape Town, South Africa; 7 Sports Performance Institute New Zealand (SPRINZ), Faculty of Health and Environmental Science, Auckland University of Technology, Auckland, New Zealand; 8 School of Sport, Exercise and Nutrition, Massey University, Palmerston North, New Zealand; 9 National Rugby League, Sydney, Australia

**Correction to: Sports Med - Open 6:6 (2020)**

**https://doi.org/10.1186/s40798-019-0233-9**

An incompletely revised version of the above article was inadvertently submitted for publication before all authors had the opportunity to approve the manuscript. As a result, multiple errors were present in the published article [1].

These relate to:

- The number of articles assessed.
- Reference citation details.
- Data errors.
- Figs 1, 2a, 2b, 3a, 3b, 4a, 4b, 5, 6a, 6b and 6c.
- Tables 1, 2, 3, 4 and 6.

The full article with all corrections is re-published ahead.
